# Intake of Spineless Cladodes of *Opuntia ficus-indica* During Late Pregnancy Improves Progeny Performance in Underfed Sheep

**DOI:** 10.3390/ani10060995

**Published:** 2020-06-07

**Authors:** Venancio Cuevas Reyes, Francisco Santiago Hernandez, Manuel de Jesus Flores Najera, Juan Manuel Vazquez Garcia, Jorge Urrutia Morales, Morteza Hosseini-Ghaffari, Alfonso Chay-Canul, César A. Meza-Herrera, Antonio Gonzalez-Bulnes, Graeme B. Martin, Cesar A. Rosales Nieto

**Affiliations:** 1 Instituto Nacional de Investigaciones Forestales, Agrícolas y Pecuarias, Campo Experimental Valle de México, Texcoco 56250, Ciudad de México, Mexico; cuevas.venancio@inifap.gob.mx; 2 Instituto Nacional de Investigaciones Forestales, Agrícolas y Pecuarias, Campo Experimental San Luis, San Luis Potosí 78431, Mexico; santiago.francisco@inifap.gob.mx (F.S.H.); urrutia.jorge@inifap.gob.mx (J.U.M.); 3 Instituto Nacional de Investigaciones Forestales, Agrícolas y Pecuarias, Campo Experimental La Laguna, Matamoros 27440, Coahuila, Mexico; flores.manuel@inifap.gob.mx; 4 Facultad de Agronomía y Veterinaria, Universidad Autónoma San Luis Potosí, San Luis Potosí 78321, Mexico; manuelvazquez87@yahoo.com.mx; 5 Institute of Animal Science, Physiology & Hygiene Unit, University of Bonn, 53115 Bonn, Germany; morteza1@uni-bonn.de; 6 División Académica de Ciencias Agropecuarias, Universidad Juárez Autónoma de Tabasco, Carr. Villahermosa-Teapa, km 25, Villahermosa 86280, Tabasco, Mexico; aljuch@hotmail.com; 7 Unidad Regional Universitaria de Zonas Áridas, Universidad Autónoma Chapingo, Ciudad Juárez 35230, Bermejillo, Mexico; cmeza2020@hotmail.com; 8 Departamento de Reproducción Animal, INIA, 28040 Madrid, Spain; bulnes@inia.es; 9 UWA Institute of Agriculture, University of Western Australia, Crawley, WA 6009, Australia; graeme.martin@uwa.edu.au

**Keywords:** birth weight, cactus, *Opuntia* spp., postnatal performance, sheep

## Abstract

**Simple Summary:**

Plants in the *Opuntia* genus are abundant and can be used as a feed supplement because they are highly digestible and can provide water and energy. We fed sheep during late gestation with alfalfa (Control), *Opuntia* (Opuntia) or protein-enriched *Opuntia* (E-Opuntia) and measured milk yield and postnatal growth in the progeny. Birth weight did not differ among treatments (*p* > 0.05) but progeny from E-Opuntia grew faster (*p* < 0.01) and were heavier at weaning (*p* < 0.05), despite the fact that Control ewes produced more milk (*p* < 0.05). Feeding ewes with *Opuntia* (protein enriched or not) during the last third of gestation is an option for reducing production costs in underfed females managed under extensive conditions in arid and semiarid regions.

**Abstract:**

The present study tested whether feeding ewes during the last third of pregnancy with cladodes of *Opuntia* (untreated or protein-enriched), as an alternative to alfalfa hay, would improve milk yield as well as the pre- and post-natal growth of their lambs. Sixty mature Rambouillet ewes and their progeny were randomly allocated among three nutritional treatments: (i) Control, fed alfalfa; (ii) Opuntia, fed untreated cladodes; (iii) E-Opuntia, fed protein-enriched cladodes (pre-treated with urea and ammonium sulphate). Birth weight did not differ among treatments (*p* > 0.05) but Control ewes produced more milk than both groups of *Opuntia*-fed ewes (*p* < 0.05). However, milk yield was not related to the growth of the progeny (*p* > 0.05) because lambs from E-Opuntia-fed ewes grew faster (*p* < 0.01) and were heavier at weaning (*p* < 0.05) than lambs from the other two groups. We conclude that *Opuntia* (with or without protein enrichment) can be used as an alternative to alfalfa hay for feeding ewes during the last third of pregnancy and therefore reduce production costs under extensive conditions in arid and semiarid regions. Moreover, protein-enriched Opuntia appears to improve postnatal lamb growth.

## 1. Introduction

The breeding of small ruminants is often the principal economic output in the arid and semiarid regions of the world. Animals raised under these conditions depend solely on the forage resources from these usually degraded rangelands, and generally do not receive any nutritional supplementation [1,2] because high-quality pastures and concentrates are not readily available and usually too expensive [3].

A possible low-cost alternative is to make use of autochthonous plants, such as cacti (*Opuntia* spp.) and ball moss (*Tillandsia recurvata*) [4,5,6]. *Opuntia* spp. offer a high water content, high digestibility and significant amounts of energy [7,8]. In cladodes of the spineless cactus, *Opuntia ficus-indica*, the protein content ranges from 2.8–8% [7,8,9,10], the fat content ranges from 3.9–4.7 g/100 g DM [9] and the content of metabolisable energy ranges from 11.1–11.4 mj/kg DM [10], with some variation according to age and genotype. *Opuntia* adapts easily to poor-quality soils, withstands water shortages and high temperatures [11,12], and is widely distributed across Latin America, South Africa and the Mediterranean region [10,13].

Previous studies have shown that feeding female sheep and goats with *Opuntia* cladodes can improve reproductive performance and the growth of their offspring post-weaning, suggesting that *Opuntia* is a viable alternative for nutritional management of small ruminants in arid and semiarid conditions [14,15,16,17]. Particularly important is the period that includes late gestation and early lactation because the female must transition from a non-lactating lipogenic status into one of high demand for energy to support the growth of fetuses and the newborn [18]. In contrast with its ability to supply energy, *Opuntia* cladodes contain low and variable amounts of protein [7,8]. This problem can be overcome to some extent by adding urea (1%) or by fermentation with various additives [17,19,20,21]. These treatments enhance the dry matter intake and digestibility of *Opuntia* [22,23]. Enhancing the nutritive value of the maternal diet during the last trimester of gestation can improve colostrum quality [24], especially when the nutritive quality of the feedstuff is low. Previous work has shown that *Opuntia* cladodes can also fill this role in sheep, increasing the production, immunoglobulin G concentration and energy content of colostrum [4,15]. However, the effect on birth weight remains unknown. Thus, in the absence of sufficient information, we tested whether feeding *Opuntia* cladodes (untreated or protein enriched), as a substitute for alfalfa hay, during the last trimester of gestation would increase milk yield and birth weight, and accelerate lamb growth.

## 2. Materials and Methods

### 2.1. Ethics Statement

The study was conducted during the breeding period on a commercial farm in northern Mexico (22°15′ N, 100°52 W). All procedures in this study are consistent with International [25] and National [26] Research Council’s Guide for the Care and Use of Laboratory Animals, with institutional approval reference number 10561934075.

### 2.2. Animals and Experimental Procedure

A total of 60 pregnant Rambouillet ewes were naturally mated and 43 of them, with the same days of pregnancy, and their progeny (33 females and 21 males) were used to investigate the effect of maternal diet during the last third of gestation (from Day 100 ± 3 of pregnancy to delivery) on birth weight and postnatal growth. Sheep were dewormed before mating with a commercially available product containing 1 g ivermectin (Baymec^®^; Bayer, Mexico). Additionally, all ewes had received an intramuscular injection of commercially available products containing 0.005 g of vitamin B12 (Catosal^®^; Bayer, Mexico), and 500,000 IU of vitamin A, 75,000 IU of vitamin D3 and 50 mg of vitamin E (Vigantol^®^; Bayer, Mexico). Sheep had free access to clean water and a block of mineral salts containing at least 17% P, 3% Mg, 5% Ca, and 75% NaCL.

**Figure 1:**
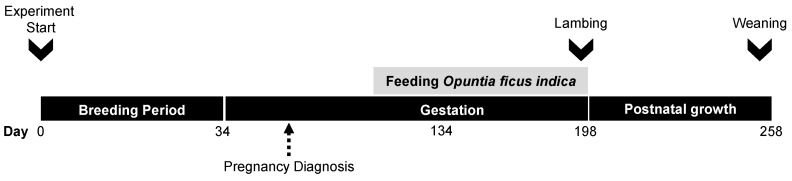
Schematic representation of the experimental design. Day 0 represents the day when the males were introduced and the breeding period started. Dietary treatments began around day 100 of gestation and continued until lambing.

The experimental protocol is shown in Figure 1. Ewes were naturally mated with trained rams for 34 days (two full reproductive cycles, mostly during August). Pregnancy, number of fetuses and gestational age were assessed three times between 30 to 45 days after the start of mating, by transabdominal ultrasonography (Samsung-Medison SA-600 fitted to a 4 MHz convex probe; Samsung Co. Seoul, South-Korea). Gestational age was estimated by assessing fetometric parameters: uterine depth (in early pregnancy), fetal crown-rump length, fetal biparietal diameter, and calcification of the fetal ribs and skull [27].

On estimated gestational day 100 ± 3, the ewes were randomly allocated among three pens, one for each dietary treatment, ensuring the average body weights of the groups were similar. The treatments were alfalfa (Control; *n* = 11; 9 singletons and 2 set of twins), untreated *Opuntia* (Opuntia; *n* = 14; 9 singletons and 5 set of twins) and protein-enriched *Opuntia* (E-Opuntia; *n* = 18; 14 singletons and 4 set of twins; see details below). Feed was provided in a fence-line feeder with sufficient space to minimize competition and allow every animal to consume its feed allocation.

### 2.3. Experimental Diets

Ewes were fed twice daily with dietary treatments from estimated gestational day 100 ± 3 up to lambing. The Control ewes were offered alfalfa hay (3% on the basis of their average body weight), whereas the ewes in the treatment groups were offered Opuntia or E-Opuntia diets. *Opuntia* cladodes were harvested daily and cut into small pieces to facilitate consumption. For the E-Opuntia treatment, cladodes were protein enriched by treating them with a solution containing 600 g urea and 80 g ammonium sulfate in 20 L of water. This solution was sprayed onto 100 kg of chopped cladodes and left for 24 h. In both *Opuntia* treatments, each ewe was initially offered 500 g per day and the amount was gradually increased to 3 kg per day. Food refusals were quantified on a pen basis but, after the two first days of the adaptation period, all of the diet offered was consumed. Therefore, on average the dry matter intake for the control treatment was 1.3 kg per animal/day and 0.78 kg per animal/day for both *Opuntia* treatments. All ewes were fed to meet their nutritional requirements but, during the last third of gestation, the amount offered was below the nutritional requirements for a dry ewe with low physical activity [28]. For instance, the diet in the control treatment was 27% above the protein requirements, but 57% below the metabolizable energy; while in the diet in the *Opuntia* treatment was 63% below the protein requirements and 35% below the metabolizable energy; whereas in the diet in the *E-Opuntia* treatment was 33% below the protein requirements and 37% below the metabolizable energy (Table 1). During early lactation, the diet was based on alfalfa, oats straw and silo to meet the nutritional requirements [28].

The nutritional composition of diets including dry matter, maintenance energy, and crude protein were assessed by AGROLAB México S.A. de C.V. (Table 1).

### 2.4. Maternal Live Weight

Ewes were weighed weekly throughout the experiment and the measurement were used to determine body weight changes.

### 2.5. Milk Yield

Before feeding, milk yield was assessed every two weeks (on the same day of the week), from one week after birth until weaning, using the oxytocin protocol to elicit milk let-down [29,30]. In brief, the ewes were separated from their lambs and then hand milked 5 min after an intramuscular injection of oxytocin (20 IU mg^−1^; PiSA Agropecuaria, Hidalgo, Mexico) according to manufacturer’s guidelines. The time of the first milking was recorded and, 3 h later, the ewes were re-milked following the same oxytocin protocol. The weight of the milk collected at the second milking and the exact time between the two milkings were recorded to allow estimation of the rate of milk yield. After the second milking, the ewes and lambs were reunited in the pen.

### 2.6. Newborn Outcomes and Offspring Growth

On the day of lambing, the date, sex, and birth weight were recorded. To measure growth rates, lambs were weighed weekly from birth until weaning (60 days). One twin-born lamb from the E-Opuntia treatment died so its weight was used only for the analysis of birth weight.

### 2.7. Statistical Analysis

Data were analyzed using SAS version 9.3 [31]. Birth weight, body weight gain and weaning weight of the progeny were analyzed using linear mixed model procedures (PROC-MIXED). Fixed effects in the model were treatment, birth type and progeny sex. Birth weight, body weight gain and weaning data were included as covariates as appropriate. Maternal body weight change and lamb body weight gain were fitted in a linear regression model of weight on time for each individual and estimates of the regression coefficients were obtained as a measure of change by unit time. Milk yield data were analyzed using a mixed linear model (PROC MIXED of SAS) with treatment as the fixed effect. For progeny birth weight, liveweight gain, and weaning weight, sex and birth type were included as independent covariates where appropriate. Sampling date was included as a repeated measure and a random effect. All two-way interactions among the fixed effects and covariates were included in each model and non-significant (*p* > 0.05) interactions were removed from the model. Significant differences among means for treatments within variables were analyzed using LSD of PROC GLM [31].

## 3. Results

### 3.1. Effects of Supplementation on Maternal Traits

**Figure 2:**
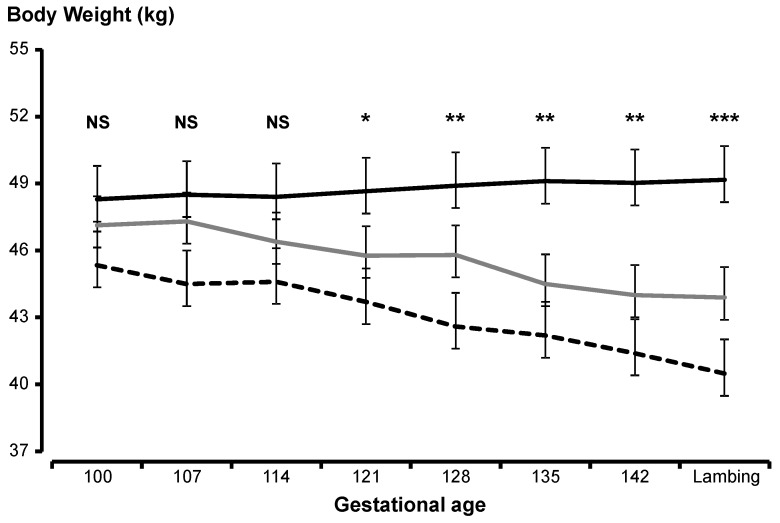
Body weight (±SEM) of Rambouillet ewes that received alfalfa (Control; grey line), untreated *Opuntia* (black solid line), or protein-enriched *Opuntia* (black dotted line) during late gestation. NS: not significant; *: *p* < 0.05; **: *p* < 0.01; *** *p* < 0.001.

As shown in Figure 2, ewe body weight did not differ among treatments at the beginning of the experiment (*p* > 0.05) but, at the end of the experiment, the differences between all treatments were significant (*p* < 0.001). The changes in ewe body weight differed among treatments (*p* < 0.001) and were −84 g day^−1^ (Control), 22 g day^−1^ (Opuntia) and −121 g day^−1^ (E-Opuntia).

**Figure 3:**
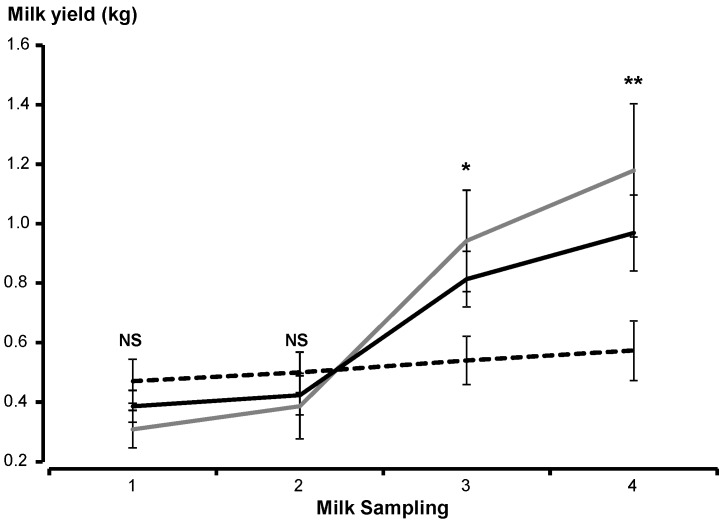
Milk yield (± SEM) from week 1 up to weaning (60 days; sampling 4) in Rambouillet ewes that received alfalfa (Control; grey line), untreated *Opuntia* (black solid line), or protein-enriched *Opuntia* (E-Opuntia; black dotted line) during late gestation. NS: not significant; * *p* < 0.05; ** *p* < 0.01; *** *p* < 0.001.

Across the experiment, milk yield differed among treatments (*p* < 0.05), sampling dates (*p* < 0.001) and the interaction between these factors was significant (*p* < 0.05). During the first two samplings after lambing, milk yield was similar among treatments and did not change with time (*p* > 0.05; Figure 3). However, differences appeared at milk samplings 3 (*p* < 0.05) and 4 (*p* < 0.01), with Control ewes producing more milk than ewes in either of the *Opuntia* treatments (Figure 3). The interactions between treatment and birth type and between treatment and progeny sex were not significant (*p* > 0.05). After combining data across all treatment groups, milk yield tended to differ between birth types (*p* = 0.07; 675 mL for singletons; 547 mL for twins) and with lamb sex (*p* = 0.06; 540 mL for females; 670 mL for males). Neither daily bodyweight gain nor weaning weight were correlated with milk yield (*p* > 0.05).

### 3.2. Progeny Birth Weight and Growth

**Figure 4:**
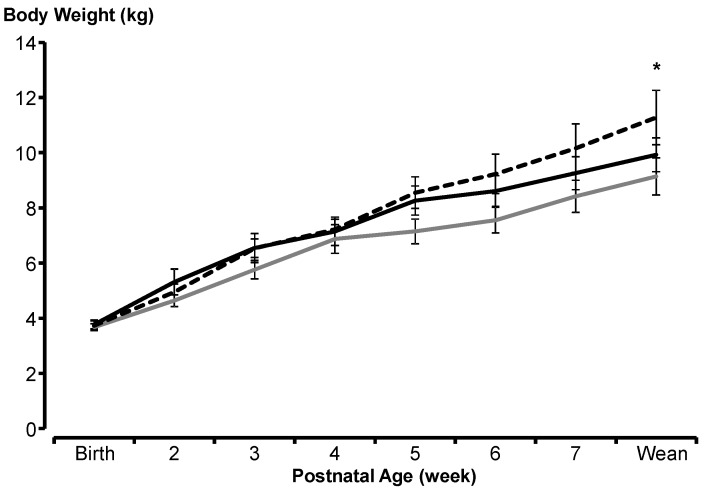
Body weight (±SEM) from birth to weaning (Week 8) in the progeny of Rambouillet ewes that received alfalfa (Control; grey line), Opuntia (black solid line), or protein-enriched Opuntia (black dotted line) during late gestation. The data are combined for birth type (singleton and twins) and sexes.

On average, the birth weight of the lambs from the CTL treatment was 3.7 ± 0.1 kg; 3.8 ± 0.2 for the lambs from the OP treatment and 3.7 ± 0.1 for the lambs from the ENR treatment. Birth weight did not differ among treatments (*p* > 0.05; Figure 4). However, weight gain during the suckling period was significantly less in the Control group (97 ± 11 g day^−1^) and the Opuntia group (97 ± 9 g day^−1^) than in the E-Opuntia group (128 ± 19 g day^−1^; *p* < 0.01), leading to significantly greater weaning weight in the E-Opuntia group (*p* < 0.05; Figure 4). Maternal milk yield was not correlated with the weight gain or the weaning weight of the progeny (*p* > 0.05).

Weaning weight differed among treatments (*p* < 0.05; Figure 4). An orthogonal contrast revealed the difference between Control and E-Opuntia to be significant for body weight on weight sampling week 4 and onwards (*p* < 0.05).

In the Control treatment, singletons (4.1 ± 0.3 kg) were heavier than twins at birth (3.5 ± 0.2 kg; *p* < 0.05). However, the difference between male (4.0 ± 0.3 kg) and female lambs from controls was not significant (3.6 ± 0.2 kg; *p* > 0.05). Daily live weight gain and weaning weights were similar between singletons and twins and between male and female lambs (*p* > 0.05).

Similarly, in the *Opuntia* treatment, singletons (4.1 ± 0.3 kg) were heavier than twins at birth (3.5 ± 0.2 kg; *p* < 0.05), whereas no difference was observed between male (4.0 ± 0.3 kg) and female lambs (3.6 ± 0.2 kg; *p* > 0.05). The daily live weight gain and weaning weights were similar between singletons and twins and between male and female lambs (*p* > 0.05).

In the *E-Opuntia* treatment, there were no differences in birth weight between sexes or between birth types (*p* > 0.05). However, in this group, male lambs grew faster (200 ± 44 g/day) than female lambs (97 ± 14 g/day; *p* < 0.05) and therefore males were heavier at weaning (15.0 ± 2.3 kg) than female lambs (9.7 ± 1.0 kg; *p* < 0.05). Daily live weight gain and weaning weight did not differ between singletons and twins (*p* > 0.05).

When all the lambs from all the treatments were combined for analysis (Table 2), the birth weights of male and females did not differ (3.8 ± 0.2 vs. 3.7 ± 0.1; *p* > 0.05). From birth to weaning, however, males grew 35% faster than females (130 ± 16 vs. 96 ± 8 g day^−1^; *p* < 0.01) and were 21% heavier at weaning (11.5 ± 0.8 vs. 9.5 ± 0.5; *p* < 0.05). In the combined groups, single-born lambs were heavier at birth than twin-born lambs (3.9 ± 0.1 vs. 3.4 ± 0.1; *p* < 0.01) and grew 27% faster (125 ± 17 vs. 98 ± 8 g day^−1^; *p* = 0.09) so were 17% heavier at weaning (11.2 ± 0.9 vs. 9.6 ± 0.5; *p* < 0.05). In the combined data for all lambs, the relationship between birth weight and body weight gain, and the relationship between birth weight and weaning weight, were not significant (*p* > 0.05). However, the weaning weight was positively related to weight gain (*p* < 0.001), with a 2.7-kg increase in weaning weight for every 50 g day^−1^ increase in weight gain.

## 4. Discussion

Feeding ewes during the last third of pregnancy with *Opuntia*, with or without protein enrichment, did not improve lamb birth weight or ewe milk yield above the levels seen with alfalfa hay. However, the E-Opuntia treatment accelerated postnatal growth and led to a heavier weight at weaning, compared to feeding either alfalfa or untreated *Opuntia*. Contrary to expectations, these were the only benefits provided by protein enrichment of *Opuntia* cladodes, perhaps because the protein content remained relatively low [32]. The impact on birth weight of *Opuntia* feeding during late gestation has been tested only a few studies, none with protein-enriched cladodes, and the outcomes generally agree with those in the present study. For example, Rekik et al. [15] supplemented Barbarine ewes during late gestation and did not observe any improvement in progeny weight at 10 days postpartum.

It is perhaps important to note that the birth weight reported in the present study was below values previously reported for this breed, at either a similar location [33] or elsewhere [34,35]. This outcome could be attributed to the amount of diet offered which, for all three treatments, did not meet the NRC [28] requirements—the Control diet provided too little energy and both of the *Opuntia* treatments provided too little protein or energy. This feeding regime was chosen to reflect the real-world situation for reproducing Rambouillet sheep in arid and semiarid regions of northern Mexico. These restrictions can explain the negative live weight gain in the ewes, as well as the lower birth weight of the lambs, in the E-Opuntia and Control treatments [36]. However, in the Opuntia treatment, the ewes did not lose weight, suggesting other factors are involved. A possible hypothesis is that the E-Opuntia intake affected rumen environment [37,38], dry matter intake and digestibility [22,23]. Late gestation is a period of high demand for energy; however, gestational nutritional restriction reduces the concentration of metabolic hormones and induces a catabolic hormonal profile [18,39,40]. Nutrient partitioning during gestation generally favors the fetus at the expense of the mother [41,42]. Hence, having in mind the previous literature, it is plausible to assume that the E-Opuntia intake increased the propionate availability in the rumen stimulating glycogenolisis and this would result in weight loss [43]. Nevertheless, fetal growth depends on nutrient availability, which in turn is related to the capacity of the placenta to transport these nutrients. Thus, maternal nutritional restriction in the current experiment may have affected fetal growth and induced a lower birth weight than the average for this breed [33,44]. The impact of *Opuntia* feeding during late gestation has been tested only by one study [15] but maternal body weights were not reported. An *Opuntia* only diet has been shown to result in weight loss in dry Rumbi ewes, whereas body weight was maintained with a combination of *Opuntia* and straw [45]. Clearly, further research is needed on the nutritional value of *Opuntia* cladodes and their ability to meet the demands of pregnancy and lactation in sheep that are underfed.

We acknowledge that, in addition to other factors [46,47,48], heavier females produce heavier offspring at birth [49,50]. However, such results were not found in the present study. Despite a lack of differences in birth weight, the lambs in the E-Opuntia group grew faster and were heavier at weaning. Interestingly, *Opuntia* supplementation has been shown to benefit post-weaning growth and fattening in other studies [14,22]. Barbarine lambs had a greater daily live weight gain when providing barley straw in combination to cactus and soybean meal or atriplex and cactus in comparison to lambs that received atriplex and barley grains [14,22]. Nevertheless, we could only find one comparable study of the impact of *Opuntia* supplementation on postnatal growth in sheep, and it was done with Barbarine ewes during early lactation. Non-protein-enriched *Opuntia* was compared to a control diets but, in contrast to the present study, both diets met the nutritional requirements and lamb growth was similar [15]. Among the other major factors affecting lamb growth in the first 8 weeks are genetic background, birth weight, the amount of milk produced by the mother, and solid feed consumption after the fourth week of age [30,51]. Milk composition is an important consideration for explaining differences in postnatal growth. Unfortunately, technical issues prevented us from analyzing milk composition, so this question awaits future studies. Previous studies have demonstrated that the growth performance of lambs is highly related to birth weight, so individuals that are heavier at birth grow faster and are heavier at weaning [30,33,52]. However, in the present study, growth rate was not related to birth weight perhaps because the birth weights were low, as discussed above. In other words, all lambs suffered fetal growth restriction and could therefore have been poorly prepared for post-natal growth to age 8 weeks.

Milk yield influences the growth of the Merino lambs [30] but, in the present study, no such relationship was evident. A similar observation was made in a previous study using only non-enriched *Opuntia* cladodes [15]. However, we expected milk yield to be improved by feeding protein-enriched *Opuntia* cladodes. The diet offered during early lactation met the nutritional requirements [28] and the milk output increased as the experiment progressed in all treatments, in a fashion typical of mature ewes [53] but, surprisingly, milk yield was greater in the Control group than in both the Opuntia and E-Opuntia groups. The high demand for energy during gestation and lactation is well known [18,54] suggesting, again, that the present observations can be explained by the effects of the low dietary energy intake on lactogenesis [24]. It seems likely that the protein content in the alfalfa, being greater than that in either of the Opuntia diets, is responsible for the better milk yield in the Control treatment [54]. In goats, milk yields have been found to be similar in control and *Opuntia*-fed animals, although the diets used in these studies contained many other ingredients that improved nutritive quality [55,56]. Nevertheless, our results indicate that, during early lactation in animals that are underfed, feeding *Opuntia* cladodes can result in moderate milk yield and perhaps reduce production costs. There is some information about how *Opuntia* feeding affects the yield and composition of colostrum and milk [15,57], but our observations suggest that research is warranted on the use of protein-enriched *Opuntia*.

The higher weaning weight with E-Opuntia, in the absence of advantages in birth weight or milk yield, might be related to prenatal programming by maternal dietary conditions. As explained above, none of our diets met the NRC requirements for pregnant ewes [28]. Underfeeding can modify fetal programming and lead to changes in metabolic pathways that affect fetal growth and postnatal fat accumulation [58]. The primary muscle fibers are established during embryonic development, with the number of fibers produced being a determinant of potential muscle mass [59,60], although fetal growth during the last third of pregnancy is due to hypertrophy [36]. Concomitantly, fetal adipogenesis begins around mid-gestation [61,62]. It appears that restriction of the maternal diet during gestation would limit the number of muscle fibers but increase the amount of central adipose tissue [63,64,65], thus compromising fetal growth and postnatal development. Indeed, in sheep, Ford et al. [64] observed that progeny from ewes that are underfed during pregnancy are heavier and with more backfat at 140 days of age, leading to the proposition of a positive relationship between intrauterine growth restriction and postnatal catch-up growth.

Finally, lambs born as singletons were heavier at birth than lambs born as twins, after which postnatal growth was similar, indicating catch-up growth by twins [66]. On the other hand, female and male lambs had similar birth weights, despite the widely accepted sexual dimorphism of the fetus [46,47]. In this case, however, postnatal growth was affected and males grew faster and were heavier at weaning than females, perhaps reflecting a more active somatropic axis in males than in females [67,68]. Overall, our results are supporting previous data on birth weight, weight gain and weaning weight between singletons and twins and between female and male lambs [30,33,48,52].

## 5. Conclusions

In conclusion, *Opuntia ficus* cladodes, with or without protein enrichment, can largely match the effects from a supplementation with alfalfa hay during the last third of pregnancy and, showing similar birth weight, milk yield and lamb growth, offer a cost-saving option for industries based in arid and semiarid regions where forage supply is limiting. Protein enrichment of the cladodes seems to have little effect on prenatal growth or milk yield, but appears to improve postnatal growth, thus accelerating weight gain in the lambs. Further research is needed to explore the impact of the supplementation of protein-enriched Opuntia on colostrum and milk composition.

## Figures and Tables

**Table 1:** Nutrient composition (DM basis) of the diets offered during the last third of gestation and nutrient requirement for a 50 kg ewe [28].

Treatment	DM (%)	CP (%)	EE (%)	CARB (%)	NDF (%)	ADF (%)	ME (Mcal/kg)
E-Opuntia	26.4	7.3	1.5	41.6	35.8	17.9	2.1
Opuntia	26.7	4.0	2.9	47.1	32.0	21.5	2.2
Control (alfalfa)	93.5	13.9	1.0	19.0	53.0	39.0	1.5
NRC requirement		10.9					3.4

DM: Dry matter; CP: Crude Protein; EE: Ether Extract; CARB: Carbohydrates; NDF: Neutral Detergent Fiber; ADF: Acid Detergent Fiber; ME: Metabolizable energy.

**Table 2:** Combined data for all the lambs from all Rambouillet ewes in all treatment groups (Control, Opuntia and E-Opuntia) showing the effects of sex and birth type on birth weight, daily live weight gain, and weaning weight.

**Variable/Sex type**	**Female**	**Male**	**SEM ^1^**	***p* Value**
*n*	33	21		
Birth weight (kg)	3.7	3.8	0.26	0.2
Live weight gain (g day^−1^)	96	130	21	0.01
Weaning weight (kg)	9.5	11.5	1.3	0.03
**Variable/Birth type**	**Singleton**	**Twin**	**SEM^1^**	***p* Value**
*n*	31	22		
Birth weight (kg)	3.9	3.4	0.27	0.002
Live weight gain (g day^−1^)	125	98	21	0.09
Weaning weight (kg)	11.2	9.6	1.3	0.3

^1^ Standard error of the mean from the mixed model output.

## Data Repository Resources

None of the data were deposited in an official repository, yet, information can be available upon request.
